# Peritumoral Enhancement for the Evaluation of Myometrial Invasion in Low-Risk Endometrial Carcinoma on Dynamic Contrast-Enhanced MRI

**DOI:** 10.3389/fonc.2021.793709

**Published:** 2022-01-17

**Authors:** Tingting Cui, Feng Shi, Bei Gu, Yanfang Jin, Jinsong Guo, Chao Zhang, Jie Ren, Yunlong Yue

**Affiliations:** ^1^ Department of MR, Beijing Shijitan Hospital, Capital Medical University, Beijing, China; ^2^ Department of Pathology, Beijing Shijitan Hospital, Capital Medical University, Beijing, China; ^3^ Department of Obstetrics and Gynecology, Beijing Shijitan Hospital, Capital Medical University, Beijing, China

**Keywords:** magnetic resonance imaging, endometrial carcinoma, uterus, dynamic contrast-enhanced imaging, risk classification

## Abstract

**Objectives:**

To explore the clinical value of subendometrial enhancement (SEE), irregular thin-layered peritumoral early enhancement (ITLPE) and focal irregular peritumoral early enhancement (FIPE) on dynamic contrast-enhanced magnetic resonance imaging (DCE-MRI) for myometrial invasion in patients with low-risk endometrial carcinoma.

**Methods:**

Seventy-seven patients with low-risk endometrial carcinoma who preoperatively underwent DCE-MRI were included. Two radiologists independently evaluated and recorded the occurrences of SEE, ITLPE and FIPE on DCE-MRI in all patients. Interobserver agreement was calculated between the two radiologists, and the relationships between SEE, ITLPE, FIPE, and myometrial invasion were analyzed based on histologic findings. For statistically significant findings, the sensitivity and specificity were calculated, and the differences in myometrial invasion evaluations were analyzed. For those with no statistical significance, images were compared with the histopathologic sections.

**Results:**

Inter-observer agreement was good (*k* = 0.80; 95%CI, 0.577–0.955) for SEE, and very good (*k* = 0.88; 95%CI, 0.761–0.972) (*k* = 0.86; 95%CI, 0.739–0.973) for ITLPE and FIPE. After consensus, SEE was identified in 12/77 (15.6%) patients; ITLPE and FIPE were found in 53/77 (68.8%) and 30/77 (39.0%) patients, respectively. SEE and ITLPE were significantly correlated with myometrial infiltration (*P* = 0.000), but FIPE were not (*P* = 0.725).The sensitivity and specificity of SEE and ITLPE for myometrial invasion in patients with low-risk endometrial carcinoma were 95.0 and 52.9%, and 85.0 and 88.0%, respectively. The area under the curve (AUC) of SEE and ITLPE for myometrial invasion were 0.740 (95%CI, 0.584–0.896), and 0.866 (95%CI, 0.763–0.970), respectively. The sensitivity and specificity were statistically different between SEE and ITLPE for the detection of myometrial invasion (*P* = 0.031, 0.016). According to the comparison between FIPE and histopathologic findings, the irregular endomyometrial junction was found in 30/77 (38.9%) cases, 24/30 (80.0%) with myometrial infiltration and 6/30 (20.0%) cases without myometrial infiltration.

**Conclusions:**

FIPE was the irregular endomyometrial junction. It can be found in patients with or without myometrial infiltration and may lead to the overestimation of myometrial invasion by SEE on DCE-MRI. ITLPE presented high diagnostic performance and specificity for myometrial invasion in patients with low-risk endometrial carcinoma.

## Introduction

Endometrial carcinoma is the most common gynecologic malignancy in women worldwide. The tumor has a global incidence of 417, 000 new cases and 97, 000 deaths in 2020 (1). The incidence of this disease in younger women has been increasing in parallel with increases in obesity, nulliparity, and polycystic ovarian syndrome (2–5). Approximately 5–30% of all reported endometrial carcinoma cases were diagnosed in younger women (6–8). For those patients, fertility preservation should be taken into consideration when deciding optimal management. Progestogen therapy might be an option in patients with low-grade endometrioid carcinoma in the absence of any myometrial invasion based on medical imaging (9, 10). Generally, the younger women diagnosed with endometrial carcinoma usually have a better outcome, because the tumor tends to present with favorable disease features, such as a favorable histologic subtype, with a lower grade lesion and minimal or absent myometrial invasion (11–16). Endometrioid adenocarcinoma (favorable histologic subtype), G1 and G2 (lower tumor grade), Stage IA (no or less than half myometrial invasion) are at low risk according to the European Society for Medical Oncology (ESMO) clinical practice guideline for risk classification of endometrial cancer (17). Information about histologic subtype and tumor grade can be acquired by curettage; however, curettage does not give information on myometrial invasion. Therefore, myometrium infiltration assessments are needed preoperatively in patients with low-risk endometrial carcinoma so that fertility-sparing progestogen therapy can be prescribed in these patients.

Magnetic resonance imaging (MRI) is considered to be a reliable modality for the evaluation of myometrial invasion of endometrial carcinoma for its excellent soft tissue contrast (18). Myometrial invasion is often assessed by previously published standards as follows: an interrupted junctional zone (JZ) on T2-weighted MR images and subendometrial enhancement (SEE) on dynamic contrast-enhanced **(**DCE) images. According to previous studies, low signal intensity JZ is the boundary between the endometrium and myometrium based on T2-weighted MR images, and SEE is the thin-layered enhancement between the endometrium and myometrium on DCE images (19, 20). However, JZ may be poorly visible due to age, menstrual cycle, acyeterion or hormone mimetics. Therefore, the diagnostic accuracy of myometrial invasion is lower if done only with T2-weighted images (21, 22). Nowadays, the diagnostic efficiency of myometrial invasion has been improved by DCE-MRI and diffusion-weighted imaging (DWI). In young women with endometrial cancer who want fertility-sparing progestogen therapy, DCE-MRI has been found superior to DWI in excluding myometrial invasion (23). With temporal and spatial resolution improvements, the sensitivity of SEE on DCE-MRI for myometrial invasion has ranged from 70 to 90%, but the specificity can be as low as 30% (24–26). The SEE is not easily detected in premenopausal patients, except during the proliferative phase of the menstrual cycle (27).This may result in a lower specificity for myometrial invasion assessments. Therefore, improving the specificity of DCE-MRI in detecting myometrial invasion in patients with low-risk endometrial cancer may be a new challenge.

Irregular thin-layered peritumoral early enhancement (ITLPE) and focal irregular peritumoral early enhancement (FIPE) were described firstly by Fujii et al. as the detailed information about the interface between endometrial carcinoma and myometrium by DCE-MRI. ITLPE was found to be related to myometrial invasion, although FIPE as a controversial finding for myometrial infiltration (26, 28). To the best of our knowledge, there are only a few publications about the diagnostic performance of ITLPE in assessing myometrial invasion in patients with low-risk endometrial carcinoma and further study of FIPE.

In this study, we aimed to assess the relationship between SEE, ITLPE, FIPE, and myometrial invasion and evaluated the diagnostic performance of SEE and ITLPE for myometrial invasion in patients with low-risk endometrial carcinoma. In addition, we compared FIPE with histopathologic findings.

## Material and Methods

### Study Population

After being approved by the Institutional Review Board and obtaining informed consents, a total of 96 consecutive patients pathologically diagnosed as endometrioid carcinoma were included at our hospital from June 2017 to March 2021. All patients underwent preoperative pelvic DCE-MRI. According to the ESMO clinical practice guidelines for endometrial carcinoma, patients with low-risk endometrioid carcinoma (2009 FIGO stage IA, G1/G2) were enrolled. The exclusion criteria were the following: 1) patients who were diagnosed by biopsy (n = 8); 2) patients who received tumor-related treatments (radiotherapy or chemotherapy) before the pelvic DCE-MRI scan (n = 4); 3) the time between DCE-MRI and surgery was >30 days (n = 3); 4) poor image quality (n = 4). Seventy-seven patients (40–77 years; mean 60 years) were eventually included in the study.

### MRI Protocol

MR examination was performed with a 1.5 T MR scanner (Ingenia, Philips Healthcare, The Netherlands) using a 32-channel phased-array body coil. All patients were asked to fast at least 4 h before the MRI examination. A series of MR sequences were performed: 1) sagittal T2-weighted imaging-turbo spin-echo (T2WI-TSE); 2) axial T2WI-TSE; 3) axial T1-weighted imaging (T1WI)-mDIXON; and 4) axial diffusion-weighted imaging (DWI). Subsequently, DCE-MRI with a flip angle of 15° was acquired. At the second dynamic, 0.2 mmol/kg of contrast agent (Gadopentetate Dimeglumine Injection, CONSUN) was administered intravenously at a rate of 2.0 ml/s and followed by the same amount of 0.9% saline flush; Twenty-five dynamics were obtained consecutively, with a temporal resolution of 7.8s, and the acquisition time was 196 s. MRI sequences and parameters are shown in **Table 1**.

**Table 1:** MRI protocol: sequences and parameters.

Sequence	Scanning plane	Repetition time (TR)/Echo time (TE) (ms)	Matrix size	Slice thickness/Gap (mm)	Field of view (mm)
T2WI-TSE	Sagittal	2,500/120	280 × 308	6/0.6	250 × 278
T2WI-TSE	Axial	3,000/110	268 × 253	4/0.5	240 × 240
T1WI-mDIXON	Axial	5.8/1.8	224 × 175	3/0	400 × 317
EPI (b = 0, 1,000 s/mm^2^)	Axial	3,659/84	144 × 110	6/0.6	400 × 300
DCE-T1WI-mDIXON	Sagittal	5.8/1.73	188 × 188	2.5/0	300 × 300

### Image Analysis

**Figure 1:**
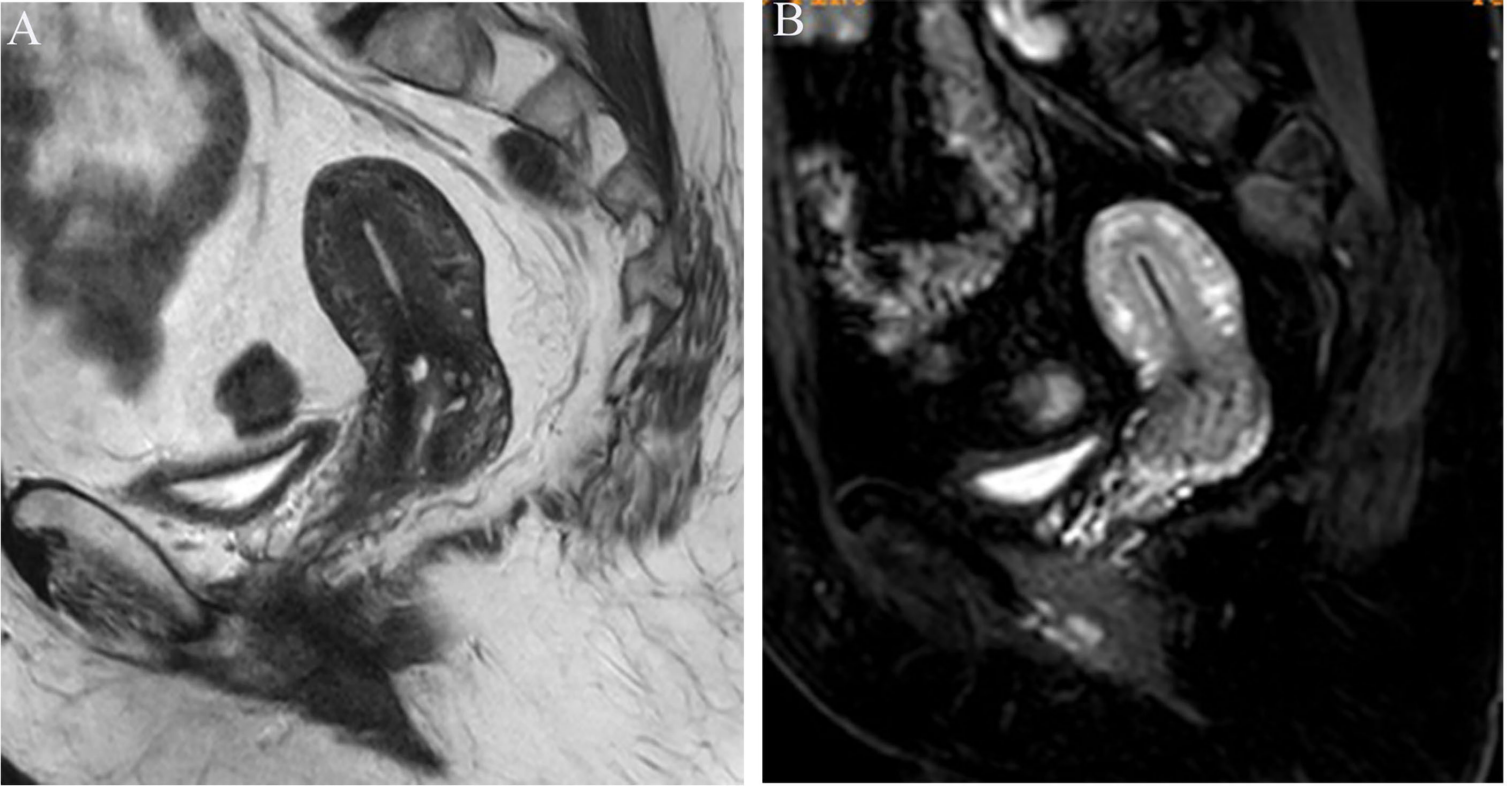
**(A)** Sagittal-T2WI image, no definitive lesion is found in the uterine cavity. **(B)** Early (25.1 s) sagittal-DCE image shows the SEE, a thin enhancement layer between the endometrium and myometrium that is regular and smooth. This case was histologically proven to have endometrioid carcinoma, G1 with no myometrial invasion.

**Figure 2:**
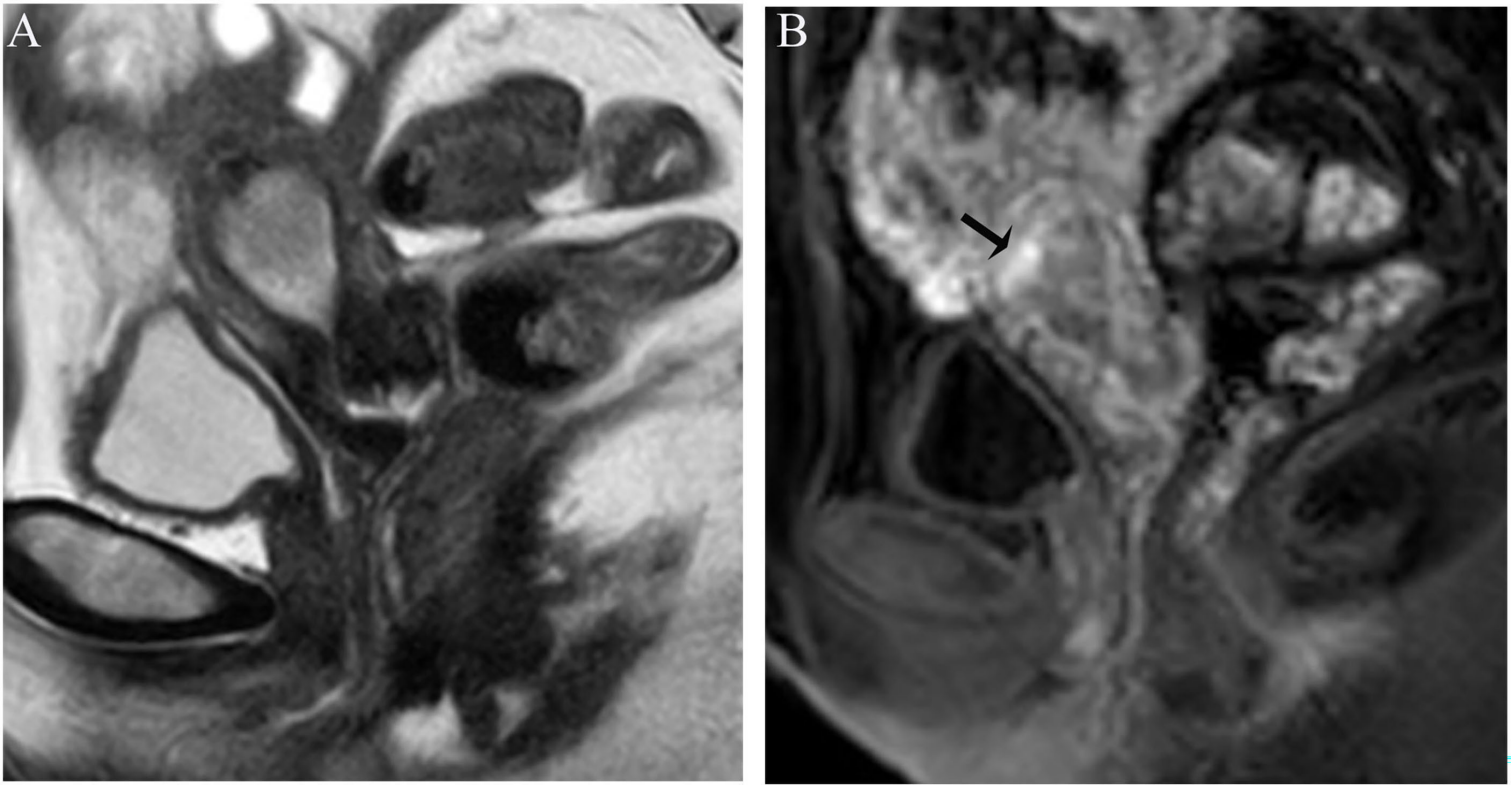
**(A)** Sagittal-T2WI image, the tumor has moderate signal intensity and is found in the uterine cavity. **(B)** Early (32.9 s) sagittal-DCE image shows an irregular thin-layered enhancement (ITLPE); an irregular thin-layered enhancement in front of the tumor (black arrow). This case was histologically proven to have endometrioid carcinoma, G2 with superficial myometrial invasion.

**Figure 3:**
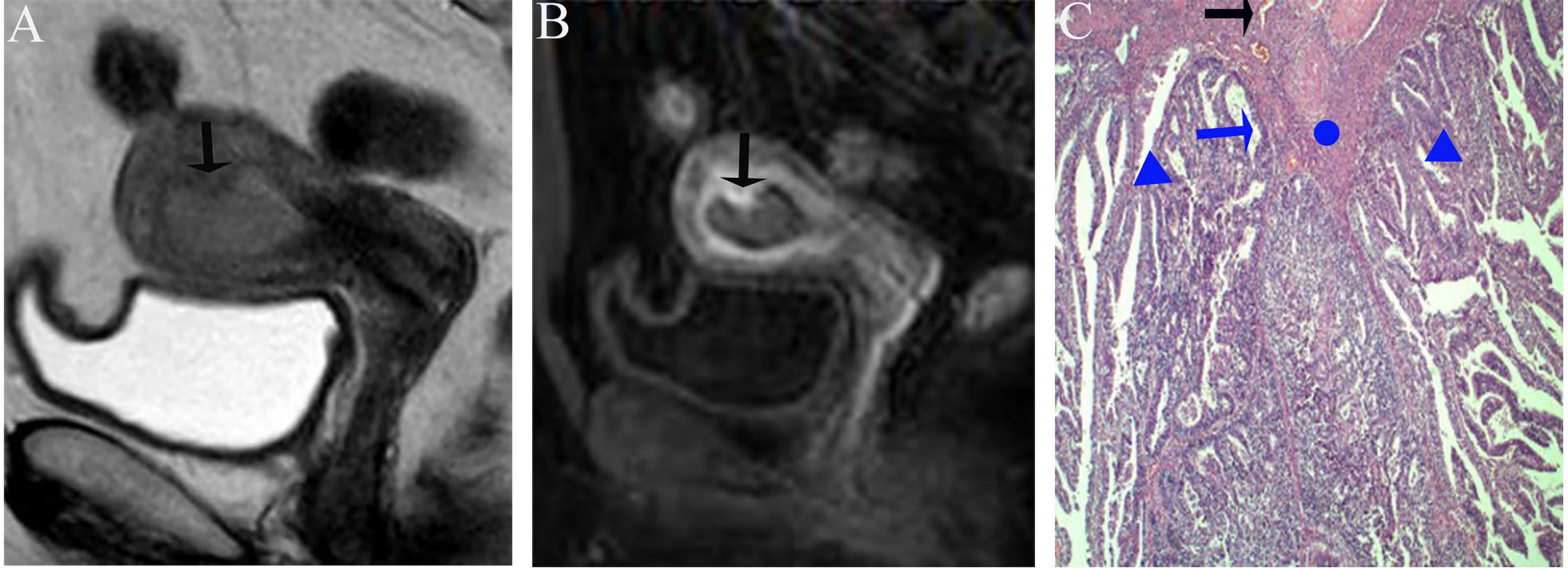
**(A)** Sagittal-T2WI image, the tumor has moderate signal intensity and is found in the uterine cavity. The focal myometrium protrudes toward the lesion (black arrow). **(B)** Early (25.1 s) sagittal-DCE image shows the FIPE, a focal irregular enhancement (black arrow). **(C)** The photomicrograph (HE, 200×) shows the irregular endomyometrial junction (blue arrow) with the dilated vessels (black arrow) of the myometrium (circle). Note the undulating contour and extension of the myometrium between the tumors (triangle). This case was histologically proven to have endometrioid carcinoma, G1 with no myometrial invasion, and was overestimated by SEE.

Image analysis was performed by two radiologists (with 20 and 25 years of experience in pelvic MRI, respectively) who were unaware of the depth of myometrial invasion (no myometrial invasion, tumor confined to the endometrium; superficial myometrial invasion, invading <50% of the myometrium; and deep myometrial invasion, invading >50% of the myometrium), tumor grade and surgical findings, except for the general diagnosis of endometrioid carcinoma. They independently evaluated and recorded occurrences of SEE, ITLPE and FIPE on DCE-MRI. Any discrepancy was resolved by consensus. Based on DCE-MRI, SEE was treated as a thin enhancement layer between the endometrium and myometrium (**Figure 1**), and was regular and smooth. According to the previous report (26), ITLPE was defined as an irregular thin-layered enhancement of the peritumoral area on early DCE images (**Figure 2**), and FIPE was the focal irregular enhancement of the peritumoral area, protruding toward the uterine cavity on early DCE images (**Figures 3A, B**).

### Histologic Analysis

All of the 77 patients underwent hysterectomy and bilateral adnexectomy with or without pelvic or para-aortic lymph node dissection. Histopathologic information, namely, histologic subtype, tumor grade, and depth of myometrial invasion, was available for all patients. The cases where FIPE were detected on images were compared with the histopathologic findings, and the histopathologic sections were reviewed by the same pathologist. The criteria for myometrial invasion and irregular endomyometrial junction followed previously published standards (29, 30). A diagnosis of myometrial invasion could be made when neoplastic epithelial cells were surrounded by myometrium without intervening endometrial stroma. Also, myometrial invasion could also be diagnosed when jagged infiltrative contour and traditional desmoplastic stromal reaction were present. The irregular endomyometrial junction was defined as an endomyometrial interface with one or more undulations that measured not less than 2 to 3 mm in magnitude.

### Statistical Analysis

Statistical analysis was performed with SPSS software (Version 22.0). Interobserver agreement between the two radiologists was calculated by the weighted Cohen’s kappa; the *k* value of 0.81–1.00 indicated very good agreement, 0.61–0.80 indicated good, 0.41–0.60 indicated moderate, 0.21–0.40 indicated fair, and 0.01–0.20 indicated poor. Analysis of the relationship between SEE, ITLPE, FIPE, and myometrial invasion based on histopathologic findings was performed with the χ^2^ or Fisher’s exact test. According to the histopathologic findings, the sensitivity and specificity of SEE and ITLPE for myometrial invasion were calculated. The diagnostic performance of SEE and ITLPE for myometrial invasion was assessed by area under the curve (AUC) of the receiver operator characteristic (ROC) curve. The differences in sensitivity and specificity between SEE and ITLPE in evaluating myometrial invasion were analyzed with McNemar’s test. A two-tailed *P*-value of <0.05 was considered statistically significant.

## Results

### MRI Findings

Radiologist 1 identified 10/77 (12.9%) patients with SEE, 55/77 (71.4%) patients with ITLPE and 27/77 (35.1%) patients with FIPE, whereas Radiologist 2 identified 14/77 (18.2%) patients with SEE, 51/77 (66.2%) patients with ITLPE and 32/77 (41.6%) patients with FIPE. Inter-observer agreement was good (*k* = 0.80; 95% CI, 0.577–0.955) for SEE, and very good (*k* = 0.88; 95% CI, 0.761–0.972) (*k* = 0.86; 95% CI, 0.739–0.973) for ITLPE and FIPE. After consensus, SEE was identified in 12/77 (15.6%) patients. ITLPE and FIPE were found in 53/77 (68.8%) and 30/77 (39.0%) patients, respectively. A statistically significant relationship was found between SEE, ITLPE, and myometrial infiltration (*P* = 0.000), but not FIPE (*P* = 0.725). The detailed information is shown in **Table 2**.

**Table 2:** Correlation between SEE, FIPE, ITLPE, and myometrial invasion.

	SEE		FIPE		ITLPE	
	(+)	(−)	(+)	(−)	(+)	(−)
Myometrial invasion						
(+)	3	57	24	36	51	9
(−)	9	8	6	11	2	15
*P*	0.000		0.725		0.000	

**Figure 4:**
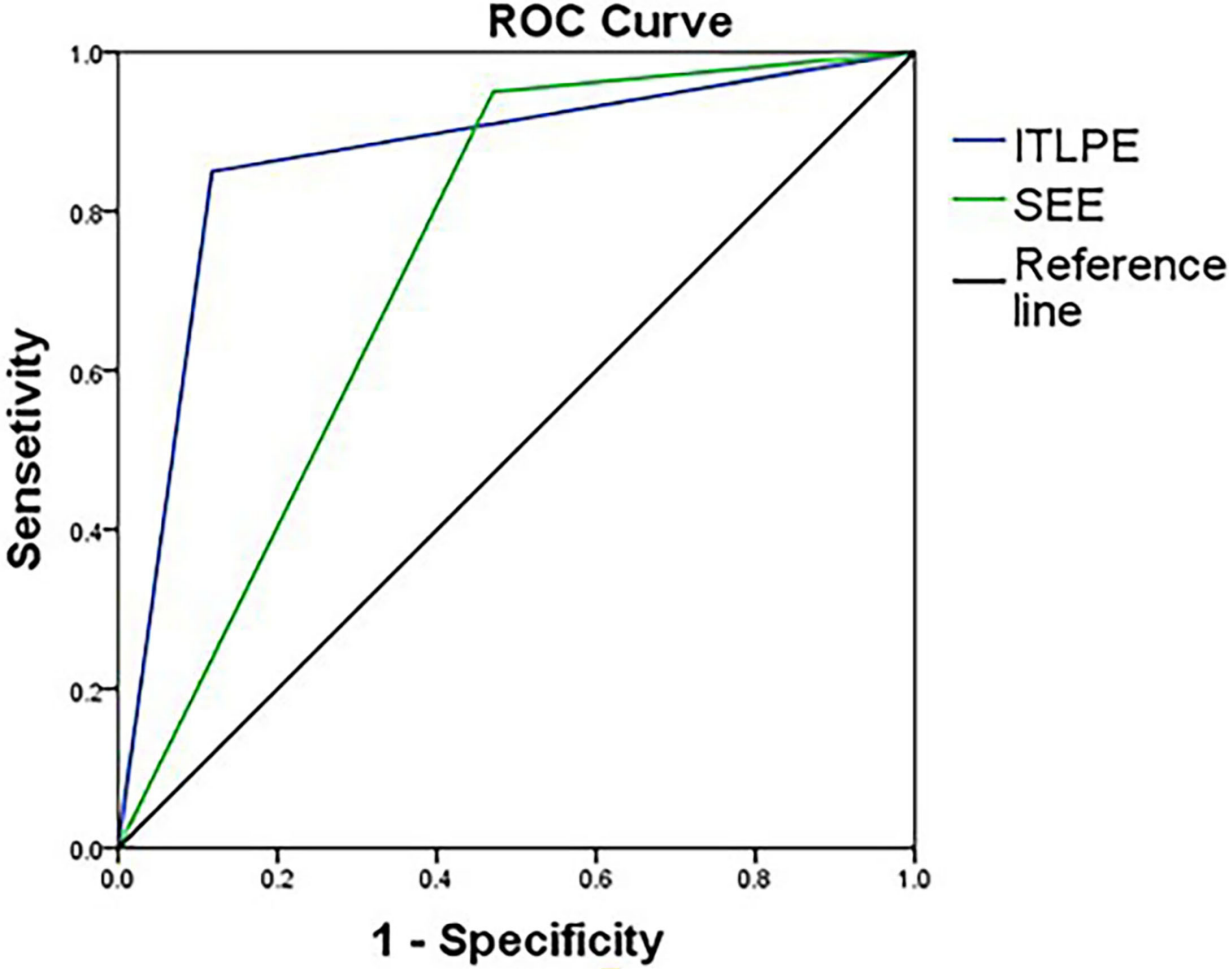
Receiver operating characteristic curve of SEE and ITLPE for myometrial invasion in patients with low-risk endometrial carcinoma.

**Figure 5:**
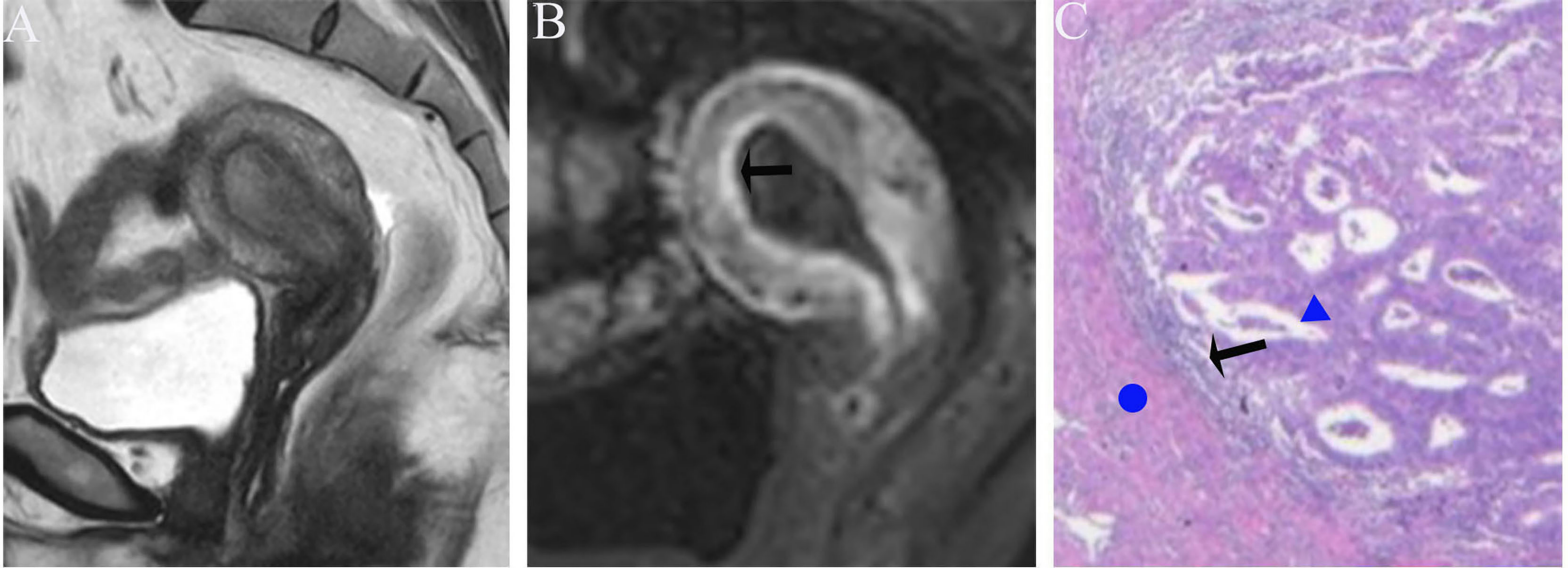
**(A)** Sagittal-T2WI image, the tumor has moderate signal intensity and is found in the uterine cavity. **(B)** Early (32.9 s) sagittal-DCE image shows that the ITLPE seem to be visible at the anterior myometrium (black arrow). **(C)** A low-power photomicrograph (HE, 40×) shows the presence of endometrial stroma components (black arrow) between the tumor (triangle) and myometrium (circle). This case was histologically proven to have endometrioid carcinoma, G2 with no myometrial invasion, and was overestimated by SEE.

**Figure 6:**
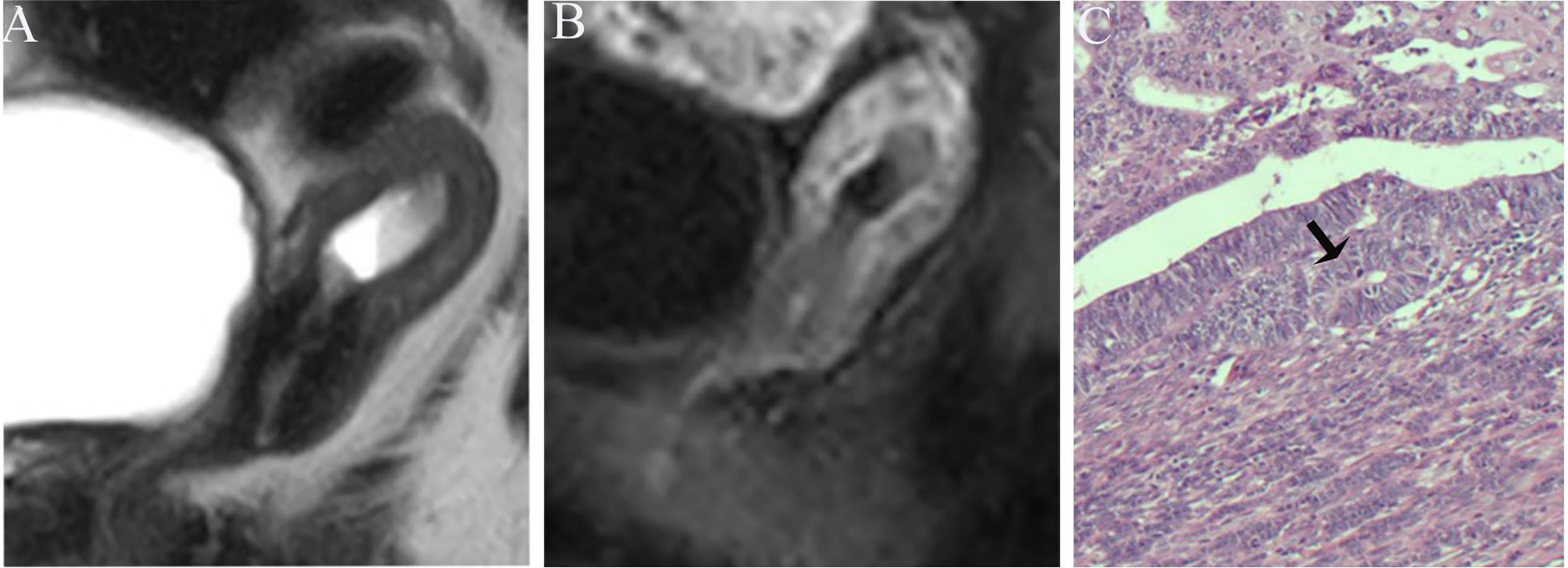
**(A)** Sagittal-T2WI image, the tumor has moderate signal intensity and is found at the bottom of the uterus. **(B)** Early (32.9 s) sagittal-DCE image shows that the ITLPE seem not to be visible. **(C)** The photomicrograph (HE, 200×) shows that the tumor is surrounded by myometrium without intervening endometrial stroma components (black arrow). This case was histologically proven to have endometrioid carcinoma, G1 with superficial myometrial invasion, and is underestimated by ITLPE.

The sensitivity and specificity of SEE and ITLPE for diagnosing myometrial invasion in patients with low-risk endometrial carcinoma are shown in **Table 3**. The AUC values of SEE and ITLPE for diagnosing myometrial invasion were 0.740 (95% CI, 0.584–0.896) and 0.866 (95% CI, 0.763–0.970), respectively (**Figure 4**). Eleven cases were misdiagnosed by SEE, 8 cases were overestimated, and 3 cases were underestimated. For the overestimated cases, SEE was recognized as incomplete by the presence of FIPE in 6 cases (**Figure 3**) and ITLPE in 2 cases (**Figure 5**). For the underestimated cases, complete SEE seemed to be visible despite the presence of myometrial infiltration. Similarly, 11 cases were misdiagnosed based on ITLPE, 9 cases were underestimated and 2 cases were overestimated. ITLPE could not be identified with or without the presence of FIPE for the underestimated cases (**Figure 6**). For the overestimated cases, ITLPE seemed to be visible despite the tumor being confined to the endometrium.

**Table 3:** Diagnostic performance of SEE and ITLPE.

n = 77	Sensitivity (%)	Specificity (%)
SEE	95.0 (57/60)	52.9 (9/17)
ITLPE	85.0 (51/60)	88.0 (15/17)
*P*	0.031	0.016

### Pathologic Findings and Comparison

The histopathologic subtype of these 77 endometrial carcinoma cases was endometrioid adenocarcinoma; 17/77 (22.1%) had no myometrial infiltration, and 60/77 (77.9%) had superficial myometrial infiltration. In all, 41/77 (53.2%) tumors were classified as grade 1 and 36/77 (46.8%) tumors as grade 2. Based on these histopathologic characteristics, all patients were classified as low-risk.

**Figure 7:**
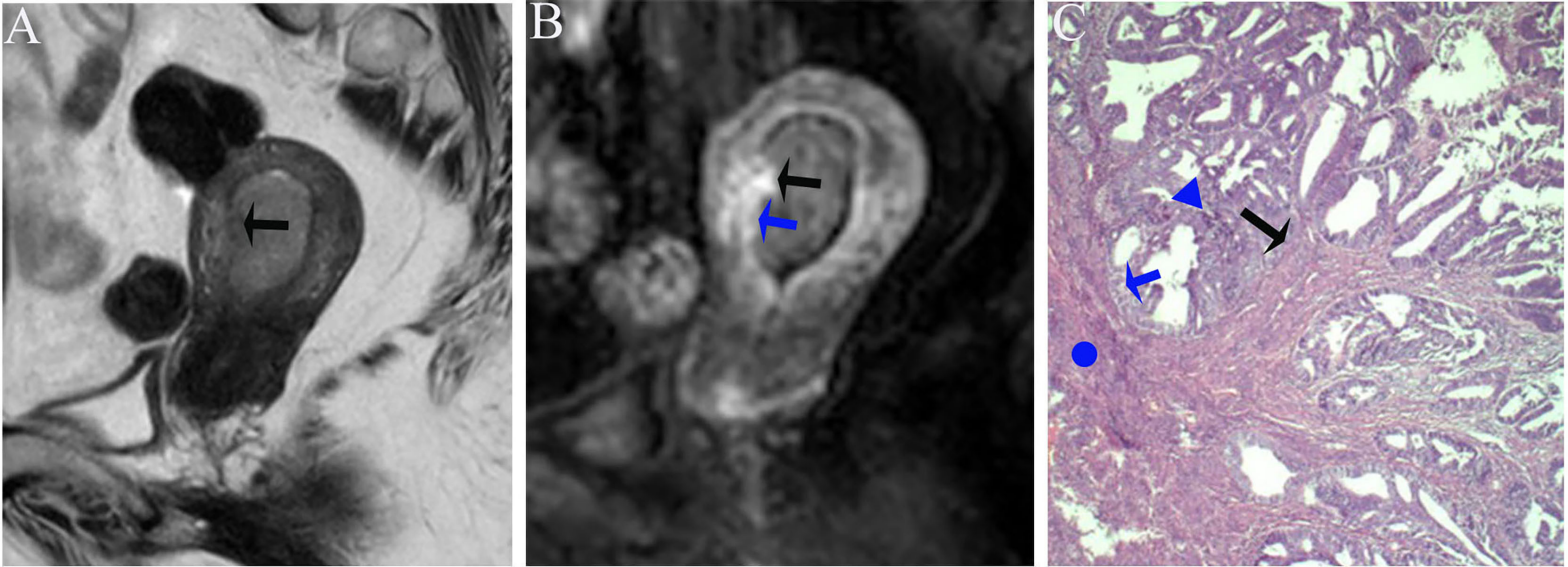
**(A)** Sagittal-T2WI image, the tumor has moderate signal intensity and is found in the uterine cavity with focal myometrium protruding toward the lesion (black arrow). **(B)** Early (25.1 s) sagittal-DCE image shows the FIPE (black arrow) and ITLPE (blue arrow). **(C)** The photomicrograph (HE, 200×) shows that the tumor (triangle) is surrounded by the myometrium (circle) without intervening endometrial stroma components (blue arrow), but the irregular endomyometrial junction (undulating contour) can be found (black arrow). This case was histologically proven to have endometrioid carcinoma, G2 with superficial myometrial invasion.

According to the comparison between FIPE and the histopathologic results, irregular endomyometrial junction can be found in 30/77 (38.9%) patients, 24/30 (80.0%) with myometrial infiltration, and 6/30 (20.0%) without myometrial infiltration (**Figures 3**, **7**).

## Discussion

Over recent years, DCE-MRI has been widely used in gynecological tumors, especially in the assessment of myometrial invasion in endometrial carcinoma (31, 32). The superior spatial and temporal resolution of DCE-MRI allowed us to observe more detailed information about the interface between the tumor and myometrium, such as ITLPE and FIPE. Our study demonstrated that the sensitivity and specificity for detecting myometrial invasion using SEE and ITLPE on DCE-MRI were 95.0, 52.9% and 85.0, 88.0%, respectively. Fujii et al. reported values of 96.6%, 32.1–46.4%, respectively, by using SEE (26). In our study, the specificity of ITLPE was higher than SEE, and higher than that of Fujii et al. In addition, in our results, the diagnostic performance of ITLPE for myometrial invasion in low-risk endometrial carcinoma was higher compared with SEE. Therefore, ITLPE that presented with high diagnostic performance and specificity maybe an efficient method to help younger patients avoid unnecessary hysterectomy. However, for the misdiagnosed cases, the main reason was that ITLPE seemed to be difficult to identify. Further temporal and spatial resolution improvements on DCE-MRI might enable more confident detection of ITLPE in future studies. Radiologists and gynecologists need the accurate identification of ITLPE to improve the diagnostic efficiency and specificity of myometrial infiltration.

The specificity of SEE for myometrial invasion was low in both our study and that of Fujii et al. The primary reason for the lower specificity was that we did not fully realize the nature of FIPE; thus, the presence of FIPE caused SEE to be misrecognized as incomplete. The comparison between the images and histopathologic findings indicated that FIPE was actually irregular endomyometrial junction caused by carcinomatous overgrowth without myometrial invasion. The irregular endomyometrial junction lent the appearance that the myometrium protruded toward the tumor with peripherally dilated vessels, which were found on the histopathologic tissue section (29). Ali et al. (30)reported that irregular endomyometrial junction was found in 57% of the surgical specimens of endometrial carcinoma. In our study, irregular endomyometrial junction was found in patients with or without myometrial infiltration, which was consistent with the study by Ali et al. In addition, our statistical analysis showed no association between FIPE and myometrial invasion. Therefore, FIPE should be taken into consideration in assessment of myometrial infiltration in low-risk endometrial carcinoma by only using SEE.

Previous reports have not recommended MRI for the surgical staging of endometrial carcinoma because of the poor-to-moderate accuracy in detecting high-risk factors, namely, deep myometrial infiltration and cervical stromal invasion (33, 34). However, ESMO, the European Society for Radiotherapy (ESTRO) & Oncology and the European Society of Gynaecological Oncology (ESGO) consensus conference on endometrial cancer (9) indicated that MRI was preferred method for detecting tumors confined to the endometrium in patients with low-risk endometrial carcinoma who might have a chance to undergo fertility-sparing progestogen therapy. In clinical practice, radiologists and gynecologists should take FIPE into account when assessing myometrial infiltration by using SEE on DCE-MRI. Then, they should look for ITLPE, which may show evidence of myometrial infiltration when an intact SEE is not detected.

There are some limitations in our study. First, the sample size is relatively small, especially regarding the patients without myometrial invasion due to its low incidence (22.1%). Secondly, the age range of patients was large (40–77 years), and some patients were postmenopausal. These factors may lead to bias in the diagnostic performance of low-risk endometrial carcinoma. Further studies should be performed in a large sample of young premenopausal patients with further improvement of the temporal and spatial resolution on DCE-MRI.

## Data Availability Statement

The original contributions presented in the study are included in the article/supplementary material. Further inquiries can be directed to the corresponding author.

## Ethics Statement

The studies involving human participants were reviewed and approved by Beijing Shijitan Hospital, Capital Medical University. Written informed consent for participation was not required for this study in accordance with the national legislation and the institutional requirements. Written informed consent was not obtained from the individual(s) for the publication of any potentially identifiable images or data included in this article.

## Author Contributions

TC, FS, and YY designed the study. CZ and JR collected the data. TC and JG analyzed the data. BG, YJ, and FS reviewed the data and interpreted the statistical analysis. TC drafted the manuscript. All authors contributed to the article and approved the submitted version.

## Conflict of Interest

The authors declare that the research was conducted in the absence of any commercial or financial relationships that could be construed as a potential conflict of interest.

## Publisher’s Note

All claims expressed in this article are solely those of the authors and do not necessarily represent those of their affiliated organizations, or those of the publisher, the editors and the reviewers. Any product that may be evaluated in this article, or claim that may be made by its manufacturer, is not guaranteed or endorsed by the publisher.

## Abbreviations

SEE, subendometrial enhancement; DCE-MRI, dynamic contrast-enhanced magnetic resonance imaging; ITLPE, irregular thin-layered peritumoral early enhancement; FIPE, focal irregular peritumoral early enhancement; ESMO, European Society for Medical Oncology; JZ, junctional zone; DWI, diffusion-weighted imaging.
